# Chemokine Levels in the Penile Coronal Sulcus Correlate with HIV-1 Acquisition and Are Reduced by Male Circumcision in Rakai, Uganda

**DOI:** 10.1371/journal.ppat.1006025

**Published:** 2016-11-29

**Authors:** Jessica L. Prodger, Ronald H. Gray, Brett Shannon, Kamnoosh Shahabi, Xiangrong Kong, Kate Grabowski, Godfrey Kigozi, Fred Nalugoda, David Serwadda, Maria J. Wawer, Steven J. Reynolds, Cindy M. Liu, Aaron A. R. Tobian, Rupert Kaul

**Affiliations:** 1 Department of Epidemiology, Johns Hopkins Bloomberg School of Public Health, Baltimore, Maryland; 2 Rakai Health Sciences Program, Kalisizo, Uganda; 3 Department of Medicine, University of Toronto, Toronto, Canada; 4 Division of Intramural Research, National Institute of Allergy and Infectious Diseases, National Institutes of Health, Bethesda, Maryland; 5 Johns Hopkins University School of Medicine, Department of Infectious Diseases, Baltimore, Maryland; 6 Department of Environmental and Occupational Health, George Washington University, Washington, District of Columbia; 7 Translational Genomics Research Institute, Flagstaff, Arizona; 8 Department of Pathology, Johns Hopkins University School of Medicine, Baltimore, Maryland; Emory University, UNITED STATES

## Abstract

Individual susceptibility to HIV is heterogeneous, but the biological mechanisms explaining differences are incompletely understood. We hypothesized that penile inflammation may increase HIV susceptibility in men by recruiting permissive CD4 T cells, and that male circumcision may decrease HIV susceptibility in part by reducing genital inflammation. We used multi-array technology to measure levels of seven cytokines in coronal sulcus (penile) swabs collected longitudinally from initially uncircumcised men enrolled in a randomized trial of circumcision in Rakai, Uganda. Coronal sulcus cytokine levels were compared between men who acquired HIV and controls who remained seronegative. Cytokines were also compared within men before and after circumcision, and correlated with CD4 T cells subsets in foreskin tissue. HIV acquisition was associated with detectable coronal sulcus Interleukin-8 (IL-8 aOR 2.26, 95%CI 1.04–6.40) and Monokine Induced by γ-interferon (MIG aOR 2.72, 95%CI 1.15–8.06) at the visit prior to seroconversion, and the odds of seroconversion increased with detection of multiple cytokines. Coronal sulcus chemokine levels were not correlated with those in the vagina of a man’s female sex partner. The detection of IL-8 in swabs was significantly reduced 6 months after circumcision (PRR 0.59, 95%CI 0.44–0.87), and continued to decline for at least two years (PRR 0.29, 95%CI 0.16–0.54). Finally, prepuce IL-8 correlated with increased HIV target cell density in foreskin tissues, including highly susceptible CD4 T cells subsets, as well as with tissue neutrophil density. Together, these data suggest that penile inflammation increases HIV susceptibility and is reduced by circumcision.

## Author Summary

The per-contact risk of infection with HIV through sexual exposure is low and highly variable. Understanding the biological basis for this variability could help in the development of new methods to prevent infection. There is some evidence that penile inflammation, even in the absence of any clinical symptoms, may increase HIV-susceptibility by recruiting CD4 T cells, the immune cell type that is the principal target of HIV. We analyzed soluble inflammatory mediators in prepuce swabs collected longitudinally from initially HIV-negative men enrolled in a randomized controlled trial of adult circumcision. We found that these inflammatory mediators were elevated in men who went on to acquire HIV. We also found that higher levels of these mediators were associated with an increased density of HIV-susceptible target cells in the underlying foreskin tissue and that circumcision reduced their levels, which may help to explain why circumcision reduces HIV risk by 60% or more. Together, these data suggest that penile inflammation, in the absence of genital infections, increases HIV susceptibility and is reduced by adult male circumcision.

## Introduction

Two million individuals acquired HIV-1 (HIV) in 2014, contributing to the nearly 37 million living with this still incurable infection [1]. While most individuals acquired the virus through heterosexual sex [2], the per act risk of female-to-male transmission is generally low (less than 1/250 per coital act in low income countries [3]). This risk is also highly variable, and is dependent on factors in both the infected and uninfected partner [4]. Susceptibility of an uninfected male partner has been epidemiologically linked to younger age [5, 6], race [7], genital co-infections [8], and lack of male circumcision [9, 10]. However, the biological mechanisms by which these parameters alter HIV susceptibility remain incompletely understood.

Mucosal inflammation and immune activation are hypothesized to enhance HIV susceptibility. In the genital mucosa, CD4 T cells expressing CCR5 are the primary targets of HIV [11–14], with potential transport and amplification by local dendritic cell subsets [15]. Thus, if inflammation leads to the recruitment of CCR5+ CD4 T cells, it will provide additional target cells for HIV. HIV also preferentially infects and replicates in activated CD4 T cells [16–21], and so augmented immune activation may also facilitate the establishment of productive mucosal infection. Contribution of mucosal inflammation to genital HIV susceptibility is consistent with data from female rhesus macaques, where pro-inflammatory cytokines promote the recruitment and activation of CD4 T cells in the vaginal mucosa [13], and the number of CCR5+ CD4 T cells at the site of mucosal challenge dictates the likelihood of subsequent productive SIV infection [22]. Furthermore, observational studies in South African women have linked pro-inflammatory genital cytokines to HIV acquisition [23] and increased CD4 T cells in the cervical mucosa [24]. While there are no similar data from men, asymptomatic herpes simplex virus type-2 (HSV-2) infection is associated with a 3-fold increased risk of HIV acquisition in heterosexual uncircumcised men [25], perhaps due to increased CCR5+ CD4 T cells in foreskin tissue [26, 27].

Randomized clinical trials have conclusively shown that male circumcision reduces HIV susceptibility in heterosexual men [28–30], but the biological mechanisms underlying this protection remain incompletely understood. One hypothesis is that circumcision reduces genital inflammation and immune activation, either through the prevention of viral STIs [31], the reduction of inflammatory anaerobic bacteria [32], or through other mechanisms yet to be defined, which in turn this reduces the density of potential target cells for HIV. This hypothesis is supported by *ex vivo* experiments demonstrating that the inner aspect of the foreskin has an increased density of HIV target cells [33–35] and more efficient virus transfer from Langerhans cells to local CD4 T cells [15] than the outer aspect, which is contiguous with the shaft skin that remains after circumcision. These observations suggest that the intact foreskin constitutes an immunologically activated tissue milieu that promotes target cell recruitment and dendritic cell maturation [36–38].

We hypothesized that elevated levels of pro-inflammatory penile cytokines would be associated with HIV acquisition in uncircumcised men and with an increased density of HIV target cells in foreskin tissue, and that cytokine levels would be reduced by circumcision. To test these hypotheses, we performed a case-control study of coronal sulcus cytokines and HIV acquisition among men who participated in a randomized controlled trial (RCT) of male circumcision in Rakai, Uganda [29]. We then examined whether these inflammatory cytokines declined after circumcision in a subset of men who were enrolled in the trial but who did not acquire HIV. Finally, we used samples from a cross-sectional study of men undergoing elective circumcision [39] to assess the correlation between prepuce cytokine levels and foreskin HIV target cell density.

## Results

### Coronal sulcus cytokines and HIV acquisition

To assess the relationship of coronal sulcus cytokines with seroconversion, we performed a nested case-control study comparing men who acquired HIV during the Rakai RCT of circumcision (n = 60, cases) to men who remained persistently seronegative (n = 120, controls). All men in this analysis were randomized to receive delayed circumcision and remained uncircumcised throughout the trial. Participant demographics are presented in Table 1. HIV seroconversion was associated with occupation, marital status, number of sex partners, condom use, alcohol consumption, and self-reported genital STI symptoms (genital ulcer, genital warts, urethral discharge), as previously reported [40].

**Table 1:** Demographics of cases and controls from Rakai, Uganda.

	Controls (n = 120)		Seroconverters (n = 60)		p-value
	No.	Col %	No.	Col %	
**Age**					
15–24	67	55.8	29	48.3	0.284
25–29	20	16.7	16	26.7	
30–49	33	27.5	15	25.0	
**Education**					
None	9	7.5	4	6.7	0.975
Primary	82	68.3	41	68.3	
Secondary+	29	24.2	15	25.0	
**Religion**					
Catholic	76	63.3	43	71.7	0.487
Protestant	36	30.0	13	21.7	
Other	8	6.7	4	6.7	
**Occupation**					
Sustenance Agriculture	38	31.7	22	36.7	0.014
Salaried Employment	8	6.7	1	1.7	
Trade/Shopkeeper	32	26.7	17	28.3	
Student	22	18.3	2	3.3	
Other	20	16.7	18	30.0	
**Marital Status**					
Single	59	49.2	27	45.0	0.004
Monogamous	55	45.8	20	33.3	
Polygamous	4	3.3	5	8.3	
Separated	2	1.7	8	13.3	
**Sex partners last 12 months**					
0	18	15.0	4	6.7	0.022
1	71	59.2	29	48.3	
2+	31	25.8	27	45.0	
**Condom use, if sexually active**					
Not using	46	45.1	16	28.6	0.027
Sometimes	31	30.4	29	51.8	
Always	25	24.5	11	19.6	
**Genital washing**					
Less than daily	23	19.2	15	25.0	0.366
Daily or more	97	80.8	45	75.0	
**Alcohol use**	79	65.8	49	81.7	0.027
**Syphilis prevalence** (n = 168)	2	1.8	4	7.0	0.109
**HSV-2 seroprevalence** (n = 164)	39	34.2	22	44.0	0.116
**Self-reported genital symptoms**	1	0.8	7	11.7	0.001

All cytokines examined were detected in coronal sulcus swabs, although many were detected infrequently. IL-8 was most common, detected in 60% of coronal sulcus swabs (concentration range >1.5–7405.7pg/ml in swabs suspended in 1ml transport medium), followed by MIG (range >0.3–6.9pg/ml), which was detected in 25% of swabs. Other cytokines, (GM-CSF, MCP-1, MIP3α, IL-1a and RANTES) were detected infrequently (<10% of participants, Table 2). Cytokine detection was not associated with sexual behavior or demographic factors (S1 and S2 Tables), but was associated with self-reported STI symptoms (genital ulcer, genital warts, urethral discharge).

**Table 2:** Association of coronal sulcus cytokines with subsequent HIV seroconversion.

	Cytokine Prevalence				Unadjusted Odds Ratio (95% CI)	*Adjusted Odds Ratio* ^A^ *(95% CI)*
	Controls		Seroconverters			
	n = 120	%	n = 60	%		
Individual Cytokines						
IL-8	63	52.5	44	73.3	2.52 (1.28, 4.99)	*2*.*26 (1*.*04*, *6*.*40)*
MIG	23	19.7	22	36.7	2.49 (1.23, 5.03)	*2*.*72 (1*.*15*, *8*.*06)*
GM-CSF	5	4.2	7	11.7	3.02 (0.92, 9.91)	
MCP-1	6	5.0	6	10.0	2.10 (0.65, 6.79)	
MIP3α	4	3.3	5	8.3	2.61 (0.68, 10.06)	
IL-1a	4	3.3	3	5.0	1.53 (0.33, 7.16)	
RANTES	3	2.5	2	3.3	1.35 (0.22, 8.30)	
Number of Cytokines^B^						
0	55	45.8	14	23.3	ref.	*ref*.
1	42	35.0	25	41.7	2.34 (1.09, 5.03)	*2*.*56 (0*.*93*, *7*.*70)*
2+	23	19.2	21	35.0	3.78 (1.61, 8.90)	*3*.*30 (1*.*21*, *12*.*50)*

^A^ Adjusted for STI diagnostics (syphilis and HSV-2), and all variables associated with either seroconversion, IL-8 or MIG (occupation, marital status, multiple sex partners, condom use, alcohol use, STI symptoms)

^B^ Number of cytokines analyzed as a categorical variable. When number of cytokines is analyzed as a continuous variable, the aOR of seroconversion = 1.42 per one cytokine increase (95% CI: 1.01–2.01).

Men who acquired HIV were more likely to have detectable levels of the chemoattractant cytokines IL-8 (aOR 2.58, 95% CI: 1.40–6.40) and MIG (aOR 3.05, 95% CI: 1.15–8.06) at the visit prior to seroconversion (Table 2). The increased odds of HIV acquisition did not change after adjusting for covariates associated with either the detection of cytokines (S1 and S2 Tables) or seroconversion (Table 1), including self-reported STI symptoms.

**Fig 1:**
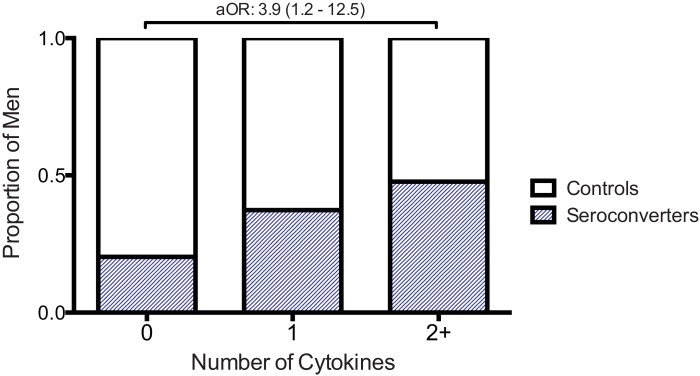
Risk of seroconversion increased when multiple coronal sulcus cytokines detected. IL-8, GM-CSF, MCP-1, MIP3α, IL-1a and RANTES were measured in coronal sulcus swabs taken at the visit immediately prior to seroconversion in men who acquired HIV (n = 60, cases). Cytokines were also measured in time-matched swabs from men who remained persistently seronegative (n = 120, controls). The proportion of men with either no, one, or two or more cytokines detected who were either seroconverters or controls is presented. Adjusted odds ratio (aOR) of being a seroconverter given two or more cytokines was detected (compared to no cytokines detected) is presented.

HIV seroconversion was not associated with the detection of other cytokines (GM-CSF, MCP-1, MIP3α, IL-1a and RANTES), but power was limited due to the low prevalence of these cytokines. However, when the total number of detectable cytokines was considered as the primary exposure, the odds of seroconversion was found to increase significantly with the presence of two or more cytokines (aOR 3.88, 95% CI 1.21–12.50; Fig 1 and Table 2).

### Impact of circumcision on coronal sulcus cytokines

**Fig 2:**
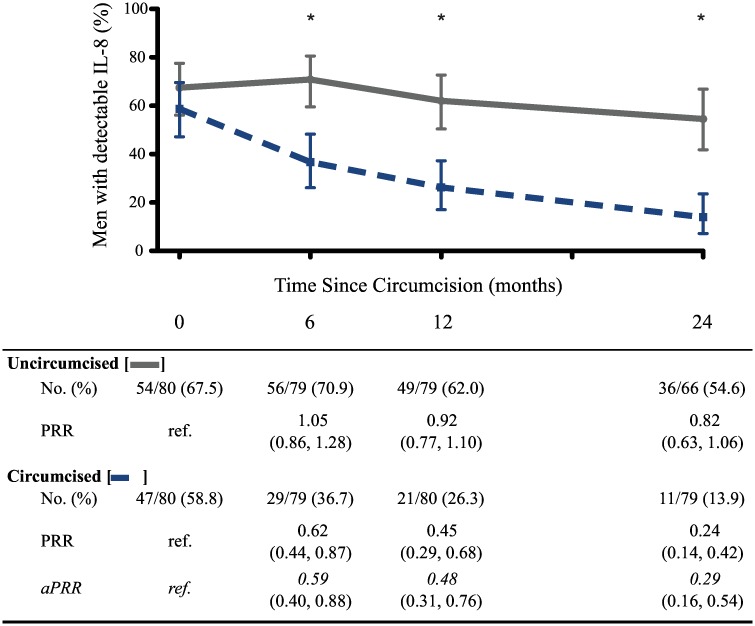
Impact of male circumcision on coronal sulcus IL-8 detection. IL-8 was measured in coronal sulcus swabs taken at enrolment (time = 0), and at month 6, 12 and 24 follow-up visits. Enrolment swabs were collected prior to surgery for men randomized to receive immediate circumcision (n = 80); control men remained uncircumcised (n = 80). The proportion of men with detectable IL-8 and 95% CIs (Fisher’s exact) is presented, with prevalence risk ratio (PRR) compared to baseline, and adjusted PRR (aPRR), below. * p-value for PRR comparing prevalence of IL-8 detection between controls and circumcised men is <0.001.

Since coronal sulcus cytokines were associated with increased HIV susceptibility, we examined circumcised and uncircumcised men who remained persistently seronegative to determine how circumcision impacts coronal sulcus cytokines levels. Enrolment demographics of men randomized to receive either immediate circumcision (“circumcised”, n = 80) or delayed circumcision (“uncircumcised”, n = 80) were similar (S3 Table). Detectable IL-8 declined significantly after circumcision (Fig 2), even though the prevalence of detectable coronal sulcus cytokines was similar between the two groups at enrollment (S4 Table). Among men who received circumcision, the prevalence of detectable coronal sulcus IL-8 declined significantly by month 6 post-circumcision (PRR month 6 compared to enrollment was 0.59, 95% CI: 0.44–0.87; Fig 2) and continued to decline throughout the 24 month follow-up period (PRR 0.29, 95% OR 0.16–0.54); the decline between months 6 and 24 was significant (PRR 0.49, 95% CI 0.25–0.96). There were no significant changes in IL-8 detection among uncircumcised men. Even though MIG was associated with seroconversion, it did not change significantly after circumcision. Likewise, the prevalence of other coronal sulcus cytokines (MIG, MCP-1, MIP3α, IL-1a and RANTES) showed no significant change after circumcision (S4 Table).

### Prepuce cytokines and foreskin T cell density

**Fig 3:**
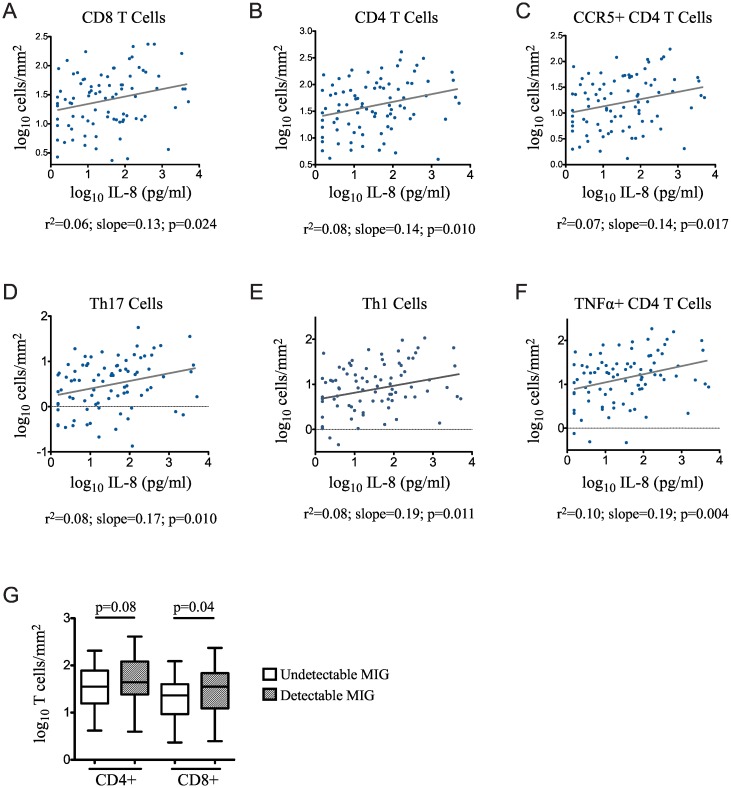
Association of cytokine levels with foreskin T cell density. IL-8 and MIG were measured in coronal sulcus swabs (n = 89) taken immediately prior to circumcision, and densities of T cell subsets were measured in foreskin tissue by flow cytometry and IHC. **A-F**: Correlation of IL-8 concentration with the following densities of T cell populations are displayed: (A) CD8+ T cells; (B) CD4+ T cells; (C) CCR5+ CD4 T cells; (D) Th17 cells; (E) Th1 cells; and (F) TNFα+ CD4 T cells. **G:** Median and range of total foreskin CD4 and CD8 T cell density stratified by detection of MIG and compared using Mann-Whitney U test. All data are log_10_ transformed.

We found that coronal sulcus IL-8 and MIG were associated with increased HIV susceptibility, and that circumcision significantly reduced IL-8. Given that IL-8 and MIG are both chemoattractant cytokines associated with recruitment of immune cells to sites of inflammation [41], we therefore examined the link between levels of prepuce cytokines and the density of pro-inflammatory and HIV-susceptible immune cell populations in foreskin tissues. We measured IL-8 and MIG levels in coronal sulcus swabs collected from 89 men who underwent elective adult circumcision at the Rakai Health Sciences Program (RHSP) Circumcision Service Program, in whom we previously characterized foreskin T cell populations [39]. Participant demographics are provided in S5 Table; no behavioral characteristics recorded correlated with levels of IL-8 or MIG. IL-8 levels were above the LLOQ in 94.4% of participants (84/89), and MIG was detectable in 51.7% (46/89). We examined the correlation of each cytokine with the density of total CD4 and CD8 T cells, and also with the following HIV target cell populations: (1) CD4 T cells expressing the HIV co-receptor CCR5 (CD3+/CD4+/CCR5+); (2) Th17 cells (CD3+/CD4+/IL-17A+); (3) Th1 cells (CD3+/CD4+/ IFNγ+); and, (4) CD4 T cells producing TNFα (CD3+/CD4+/ TNFα+). IL-8 concentration correlated with the density of both CD4 and CD8 T cells (Fig 3A and 3B; p<0.05), and with the density of CD4 T cell subsets known to be preferential HIV target cells: CCR5+ CD4 T cells, Th17 cells, Th1 cells, and TNFα+ CD4 T cells (Fig 3C, 3D, 3E and 3F; all p≤0.02). Having detectable coronal sulcus MIG was only associated with a non-significant trend of increased total CD4 (44.0 vs. 33.5 cells/mm^2^, p = 0.08; Fig 3G), but with a significant trend to increased CD8 T cell density (35.5 vs. 22.7 cells/mm^2^, p = 0.04).

**Fig 4:**
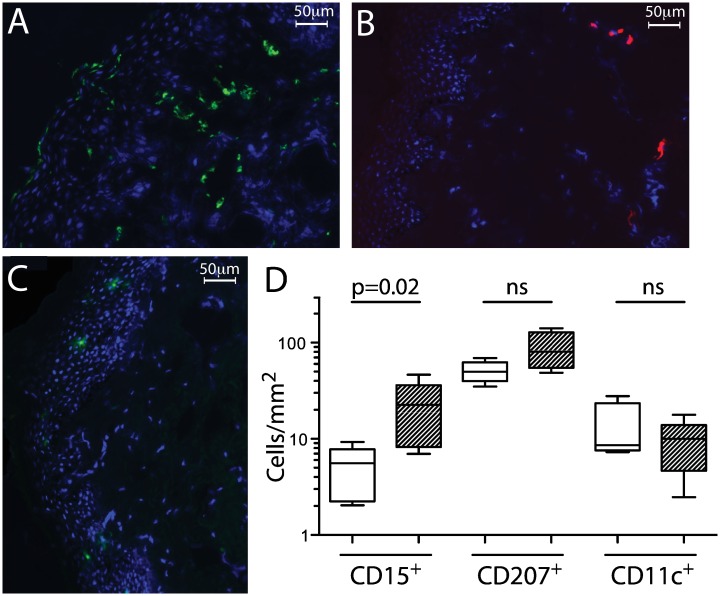
Association of cytokine levels with foreskin neutrophil and dendritic cell density. Immunofluorescence was used to measure neutrophil and dendritic cell densities in foreskin tissues from men with either high (n = 5) or low (n = 5) coronal sulcus IL-8 and MIG levels. **A-C**: Representative images of: (A) CD15+ neutrophils (green); (B) CD11c+ dermal dendritic cells (red); and (C) CD207+ Langerhans cells (green). Nuclei are counterstained with DAPI (blue) in all images. **D:** Median and range of foreskin neutrophil density in total foreskin tissue, Langerhans density in epidermal tissue, and dermal dendritic cell density in dermal tissue, from men with high (hatched bars) or low (open bars) cytokine levels. Groups compared using Mann-Whitney U test.

To investigate the relationship of other tissue immune cell populations with prepuce cytokine levels, we next assessed neutrophil (CD15+) and dendritic cell (CD207+ Langerhans and CD11c+ dermal dendritic cell) density in a subset of men with high (n = 5; median IL-8 = 3422.6pg/ml, all MIG detectable) and low (n = 5; median IL-8 = 1.8pg/ml, all MIG undetectable) coronal sulcus cytokine levels (Fig 4A–4C). Neutrophils were found in both the epidermis and dermis, often in concentrated foci; Langerhans cells were found almost exclusively in the epidermis, and CD11c+ cells were predominantly in the dermis, as previously reported [42]. Men with high prepuce cytokine levels had a 4-fold higher density of tissue neutrophils than men with low cytokine levels (22.6 vs. 5.6 cells/mm^2^, p = 0.016; Fig 4D). However, densities of dendritic cell populations were similar in men with high and low cytokine levels (CD11c: 10.0 vs. 8.6 cells/mm^2^ dermal tissue, ns; CD207: 80.6 vs. 49.8 cells/mm^2^ epidermal tissue, ns).

To rule out potential confounding of coronal sulcus cytokine levels by the vaginal secretions of a female sexual partner, cytokines were also assessed in female partner vaginal swabs collected on the day of male circumcision [43] for all 89 men in this analysis. Both IL-8 (median 699.5pg/ml, range 1.5–5693.9pg/ml) and MIG (median 5.9pg/ml, range 0.3–853.4pg/ml) were detectable in vaginal swabs, but we found no correlation between vaginal and penile cytokines within couples (Spearman’s rho: IL-8 = 0.17, MIG = 0.14, both not significant), suggesting that cytokines detected in coronal sulcus swabs did not originate from the female partner.

## Discussion

Our study demonstrates a significant link between pro-inflammatory coronal sulcus cytokines and HIV acquisition in heterosexual men. Specifically, the chemoattractant cytokine IL-8 was associated with both an increased odds of seroconversion and an increased density of highly-susceptible HIV target cells in the foreskin. In addition we found that male circumcision, a procedure that significantly reduces HIV acquisition, progressively reduced detection of coronal sulcus IL-8 during two years of follow-up (PRR of 0.29 at 24 months post-circumcision). Overall, these results suggest that the protective effect of male circumcision against HIV may be mediated in part through reductions in genital inflammation and the subsequent inflammation-mediated recruitment of HIV-susceptible cells to the foreskin. Although the mechanism(s) underpinning the relationship between cytokines and HIV susceptibility could not be fully elucidated by this observational study, the observation that coronal sulcus IL-8 and MIG were associated with HIV seroconversion is in keeping with a recent report that pro-inflammatory vaginal cytokines in women predict HIV acquisition [23], and with *in vitro* experiments demonstrating that IL-8 increases HIV susceptibility in cervical explants [20].

Both IL-8 and MIG belong to the chemokine family, a group of structurally similar small molecules that act as chemoattractants for immune cells expressing appropriate receptors. Since HIV predominantly infects CD4 T cells [11, 13, 44], both the availability and the HIV-permissivity of local CD4 T cells may dictate whether or not infection is established, with a limited number of highly susceptible cells driving initial mucosal infection [45, 46]. Therefore, recruitment or activation of specific subsets of CD4 T cells that are especially permissive to HIV may be important; Th17 and Th1 cells are highly HIV-permissive *in vitro* and are preferentially depleted *in vivo* during acute infection [47–50], and Th17 cells have recently been shown to be the primary targets of SIV, representing 64% of infected cells 48 hours after vaginal challenge [44]. Furthermore, men who are regularly HIV exposed but remain seronegative (HESN men) have a decreased relative abundance of both Th17 and TNFα+ CD4 T cells in their foreskin tissue [43]. Our finding that detectable coronal sulcus IL-8 was significantly associated with an increased overall number of CD4 T cells, including an increased density of highly susceptible Th17, Th1 and TNFα+ CD4 T cells, suggests that target cell availability may contribute to the association between coronal sulcus chemokines and HIV susceptibility.

While the association between foreskin IL-8 and HIV target cells does not prove this cytokine recruits or is produced by HIV target cells, a causal relationship is plausible. Epithelial cells (including keratinocytes) and macrophages responding to early immune stimuli are the main source of IL-8 during skin inflammation [51–54]. While many cell types express the IL-8 receptors CXCR1 and CXCR2 [55], IL-8 primarily recruits and activates neutrophils [51, 56, 57], which is consistent with our data showing a 4-fold higher density of neutrophils in foreskin tissues of men with high prepuce IL-8 levels. Neutrophils recruited by IL-8 are activated by bacterial antigens in the presence of inflammatory cytokines (IFNγ) to produce Th17-recruiting chemokines (MIP-3α and MCP-1) and Th1-recruiting chemokines (MCP-1 and IP-10) [58–60]. In turn, Th17 and Th1 cells produce IL-17 and IFNγ, respectively [61], both feeding back into the inflammatory cascade: IL-17 stimulates epithelial cells to produce more IL-8 [62], and IFNγ contributes to neutrophil chemokine production [60, 63]. Reciprocally, Th17 cells may directly contribute to IL-8 levels: Th17 cells have recently been shown to be the only subset of CD4 T cells to produce high levels of IL-8 [60, 64, 65]. In support of this, treatment with antibodies preventing the formation and action of Th17 cells (secukinumab, specific for IL-17A/IL-23) prevents neutrophil recruitment to the skin and keratinocyte immune activation, and reduces local levels of IL-8 [66]. Thus, IL-8 may be part of a positive-feedback loop, whereby local innate immune cells recruit neutrophils through IL-8, which in turn recruit HIV target cells through MIP-3α and MCP, and these HIV target cells then produce inflammatory cytokines, feeding back into local innate immune cell activation and IL-8 production. We hypothesize that this positive-feedback loop provides a causal basis for the association that we observed between prepuce IL-8 levels and HIV target cells, and that HIV target cells recruited by this mechanism drive the observed increased risk of seroconversion.

HIV seroconversion in our cohort was also associated with the detection of MIG and was increased if multiple cytokines were present. MIG is produced by macrophages in response to IFNγ and directly recruits activated T cells through CXCR3 [67], consistent with our observations that men with detectable MIG had increased numbers of foreskin CD8 T cells and a trend towards increased CD4 T cells (p = 0.08). The recruitment of IFNγ-producing T cells by MIG may also feed into to the relationship between IL-8 and HIV target cell recruitment, since IFNγ contributes to neutrophil activation and Th1/Th17 cell recruitment (as above). Of note, MCP-1 and MIP-3α were the next most abundant cytokines detected in this study (after IL-8 and MIG), and these two chemokines have been shown to specifically mediate the recruitment of Th17 cells by neutrophils [60]. We therefore hypothesize that increased risk of seroconversion with multiple cytokines is due to MIG, MCP-1 and MIP-3α feeding into the local inflammatory feedback loop described above. However, while this observational study provides important *in vivo* human data, further *in vitro* or animal studies will be necessary to completely define the causal nature of these associations.

Other cell types, such as Langerhans cells and dermal dendritic cells have been shown to facilitate HIV infection in the foreskin [68], and may contribute to increased HIV-susceptibility observed in this study. While tissue density of Langerhans cells and dermal dendritic cells was not associated with prepuce IL-8 or MIG, local tissue inflammation may contribute to dendritic cell maturation and function, thereby facilitating HIV transfer to susceptible T cell populations, as has been previously described [68, 69].

The reduction in coronal sulcus chemokines after male circumcision sheds light on a potential biological mechanism by which circumcision protects against HIV acquisition: reduced penile inflammation. We observed a significant decrease in coronal sulcus IL-8 at the first follow-up visit after circumcision (PRR = 0.59 at 6 months, 95% CI: 0.40–0.88), and IL-8 continued to decline up to study conclusion (PRR = 0.29 at 24 months, 95% CI: 0.16–0.54), which was significantly lower than at month 6. This may reflect a gradual decline in HIV target cells within remaining penile tissue, as effector T cells have been shown to be slow to clear a site of previous infection, and are enriched in tissue sites for months after antigen becomes undetectable in the skin [70]. *In vitro* studies also suggest that target cell availability contributes to the efficacy of male circumcision, as the inner aspect of the foreskin contains a comparatively high density of HIV target cells [15, 33–35]. Male circumcision may reduce HIV susceptibility by reducing penile inflammation and HIV target cell availability.

The factors causing penile inflammation could not be fully delineated in this study. Prepuce cytokine levels in uncircumcised men do not correlate with urethral cytokines (Kaul R and Galiwango R; unpublished), and we found no association with female partner vaginal cytokines, suggesting that prepuce cytokines do not derive from these sources, and may be produced in foreskin tissue. No significant associations were observed between IL-8 or MIG and age, number of sex partners, condom use, genital washing or seroprevalent STIs (HSV-2 and syphilis). While self-reported STI symptoms were associated with increased detection of both IL-8 and MIG, this did not fully account for the association between these cytokines and seroconversion. Additionally, even in symptom-free participants uninfected by HSV-2 and syphilis, we observed IL-8 concentrations ranging over 1000-fold (>1.5 to 2626.9pg/ml), suggesting that “normal” penile immune parameters are highly variable, and may be affected by factors other than classical STIs. This is in agreement with findings in South African women, where vaginal chemokine levels were associated with increased risk of HIV acquisition, but were incompletely explained by the presence of STIs [23]. Determining the factors contributing to heterogeneity in genital inflammation warrants further research as prior simulation studies have shown that variability in HIV susceptibility can affect HIV epidemic dynamics significantly and may explain differences in HIV epidemic trajectories between populations [71].

One possible contributor to penile inflammation is the resident microbiome. Alterations in the vaginal bacterial community in women, such as bacterial vaginosis, are associated with increased risk of HIV [72–75], possibly due to local inflammation [76, 77]. Th17 cells are essential in the defense against bacterial infections [78, 79], and colonization by pathogenic bacteria may increase HIV susceptibility by increasing Th17 cell density [50]. We have previously found that uncircumcised men are more likely to have BV-associated anaerobic bacteria in their sub-preputial space [80], and that circumcision gradually reduces both the total bacterial load and the abundance of these anaerobes [32]. Of note, while anaerobe abundance decreased rapidly within 6 months post-circumcision, it continued to decline for up to 24 months. This is similar to the gradual decline in IL-8 levels that we observed over the same period, and so the role of the penile microbiome as a driver of tissue inflammation and HIV susceptibility may be an interesting area for future study.

A limitation of the current work was the low concentration of cytokines in coronal sulcus swabs, especially swabs collected during the circumcision RCT. Swabs from the RCT were stored at -80°C for up to 10 years between collection and cytokine analysis. Cytokines, including IL-8, have been shown to degrade after 4 years, despite ideal storage conditions [81]. This likely explains the difference in detectability of IL-8 between swabs collected during the RCT and those collected from men attending the Circumcision Service Program, as swabs from the latter group were analyzed within one year of collection. However, it is unlikely that IL-8 degradation can account for differences observed between comparator groups in this study. In the case control study of HIV seroconversion, controls were matched to cases based on visit (time) and swab storage time did not vary between groups (median 4.7 years for both groups). Additionally, we found no correlations between IL-8 concentration and date of swab collection, suggesting that variability in swab storage time due to the relatively short duration of the trial (August 2003- November 2006) cannot account for the differences in IL-8 levels observed when examining the impact of circumcision on cytokine levels. It is possible that associations with other cytokines may have been missed due to low analyte concentration, explaining why cytokines observed to be released *ex vivo* from foreskin explants were not detectable in swabs in this study [33, 35, 69].

In conclusion, penile inflammation is an important risk factor for HIV acquisition in heterosexual men. HIV acquisition was associated with elevated levels of coronal sulcus IL-8 and MIG, which correlated with an increased density of T cells in the underlying foreskin tissue. In particular, prepuce concentrations of IL-8 were correlated with both an increased overall tissue density of CD4 T cells, as well as an increased density of specific highly HIV-susceptible CD4 T cell subsets. Finally, circumcision progressively reduced coronal sulcus IL-8 for up to 24 months after the procedure, which suggests a reduction in penile inflammation may be one mechanism by which circumcision is protective against HIV. Identifying causes of penile inflammation and immune activation in otherwise healthy men may lead to novel interventions to reduce the sexual transmission of HIV.

## Materials and Methods

### Study participants

We examined samples and data collected from two study populations enrolled through the Rakai Health Sciences Program (RHSP) in Uganda: one enrolled in an RCT of male circumcision, conducted from 2003–2006 [29]; and the second enrolled in an observational cross-sectional study through the RHSP Circumcision Service Program between 2010–2011 [39]. Study design and sample population selection are described in detail in the Statistical Methods Section, below.

#### Study participants: Circumcision RCT

A total of 4,996 uncircumcised HIV-seronegative men aged 15–49 were enrolled in the RCT of male circumcision [29]. Men were randomized to receive either immediate circumcision or circumcision delayed for 24 months, and were followed up at 6, 12 and 24 months to detect incident HIV infection and STIs, and to monitor sexual risk behaviors. At enrollment, all consenting men provided interview information on sociodemographic, behavioral and health characteristics and underwent a physical examination. Men with evidence of genital infections (urethral discharge, genital ulceration or dysuria) were treated and reassessed to ensure resolution of infection before surgery. Symptomatic STIs at follow-up visits were treated, but study visits were not delayed until resolution of symptoms. A detailed interview on time-varying behavioral and health characteristics was performed by a clinical officer at all follow-up visits.

In addition to collecting data on self-reported genital symptoms, blood samples were obtained at all visits for HIV, syphilis and HSV-2 serology. The rapid plasma reagin test (RPR test, Becton Dickinson, Franklin Lakes, NJ USA) was performed to screen for syphilis, and positive samples confirmed by Treponema pallidum particle agglutination assay (Serodia TP-PA kit, Fujirebio Diagnostics, Malvern, USA). Testing for HSV-2 was performed using an HSV-2 IgG enzyme immunoassay (Kalon Biological, Guildford, UK) using cutoffs previously validated in Rakai [82]. HIV status was determined by two enzyme immunoassays (EIAs); Vironostika HIV-1/2 Plus O (Organon Teknika, Charlotte, NC, USA) and Murex HIV-1.2.0 (Murex Biotech Limited, Dartford, UK), run in parallel. All samples discordant on the two EIAs and seroconverters were subjected to Western blot confirmation (HIV-1 Western Blot; Bio-Merieux-Vitek). All first-time positive HIV results were confirmed by the detection of HIV RNA in serum using PCR and, in the case of seroconversions, serum from the visit prior to seroconversion was screened for HIV RNA using PCR to rule out acute infection. RNA was extracted using the Abbott Sample Preparation System, and amplification was performed using the Real Time HIV-1 Amplification Reagent Kit (Abbott Molecular, Des Plaines, IL) and run on the M2000rt (Abbott) with a lower limit of detection of 40 copies/ml.

#### Study participants: Circumcision service program

To assess the relationship between prepuce cytokines and foreskin T cell density, we retrospectively analyzed all swabs collected from HIV-negative men (n = 89) enrolled in a cross-sectional, observational study of men presenting for elective adult circumcision at the Rakai Circumcision Service Program [26, 39, 43, 83]. During the observational study, men were enrolled at their surgical visit, and consented to provide information on socio-behavioral characteristics and underwent a physical examination. The primary female partner of each man was also offered participation, and if she consented, provided a self-collected vaginal swab. Men with evidence of genital infections (urethral discharge, genital ulceration or dysuria) were treated and reassessed to ensure resolution of infection before surgery. Blood samples from men were obtained for HIV and HSV-2 serology, using the same methodology as during the RCT. Male participants were also screened for acute HIV infection using real time PCR.

#### Study participants: Ethics statement

All participants provided written informed consent, and ethical approval for both new studies and assays on stored samples was obtained through Institutional Review Boards at the University of Toronto, the Uganda Virus Research Institute Scientific Ethics Committee, and the Johns Hopkins University Bloomberg School of Public Health. All samples were anonymized.

### Sample collection

Dacron swabs moistened with sterile saline and rotated twice around the full circumference of the penis at the coronal sulcus were collected from all men at enrollment and each follow up visit during the RCT, and once, immediately prior to circumcision, from the Circumcision Service participants. The same clinical officers collected swabs throughout both studies and care was taken to collect each swab in a consistent manner. Female partners of Circumcision Service Participants were asked to insert a Dacron swab into the vagina, rotate once, and remove it. Swabs were immediately placed in 1ml undiluted AMPLICOR STD Specimen Transport Kit medium (Roche Diagnostics, Indianapolis, IN) at 4°C for less than 4 hours, and then suspended, aliquoted and stored at −80°C. Foreskin tissue removed during circumcision was also collected from Circumcision Service Participants. Tissue was processed immediately upon surgical removal: two sections from distal locations on the foreskin (one from the approximate center of the inner aspect, and one from the center of the outer aspect) were snap frozen into cryomolds in Optimal Cutting Temperature (OCT) compound (both Fisher Scientific, Toronto, Canada) for immunohistochemistry; and one large section containing equal area of the inner and outer aspects reserved for T cell isolation.

### Cytokine measurement

An electrochemiluminescent detection system using a custom Human Ultra-Sensitive kit from Meso Scale Discovery (Rockville, MD) was used to assay cytokines in coronal sulcus swabs from both RCT and Service Program participants. Cytokines assessed were: IL-1α (interleukin-1α), IL-8, MCP-1 (monocyte chemotactic protein-1), MIG (monokine induced by γ-interferon), MIP-3α, RANTES (Regulated on Activation, Normal T cell Expressed and Secreted), and GM-CSF (granulocyte macrophage colony-stimulating factor). Samples from each of the three analysis sets (Coronal sulcus cytokines and HIV acquisition, Impact of circumcision on coronal sulcus cytokines, and Prepuce cytokines and foreskin T cell density) were run on plates from the same manufacturer’s lot, with samples from participant groups in each analysis set (i.e. cases and controls, circumcised and uncircumcised) distributed randomly and evenly proportioned across plates. Samples were run in duplicate, and results with a coefficient of variation (CV) above 20% for the two wells were re-run. An internal biological control was run in at two concentrations in duplicate on every plate to monitor plate-to-plate variability: internal control was made from pooled mucosal samples from 5 donors, with any low level analytes augmented by adding the relevant recombinant human protein. This sample was aliquoted for single use, and run both neat and diluted 1/20 on each plate (for biological and low-concentration controls). Plates were re-run if the concentration of any analyte in the internal control was >3 standard deviations different from the average concentration from that analyte for the first 5 plates run. Plates were imaged using the Sector Imager 2400A platform (Meso Scale Discovery). The study lower limit of quantification (LLOQ) for each analyte were as follows: IL-1α = 0.6pg/ml; IL-8 = 1.5pg/ml; MCP-1 = 0.6pg/ml; MIG = 0.3pg/ml; MIP-3α = 3.0pg/ml; RANTES = 0.6pg/ml; and GM-CSF = 0.3pg/ml. Cytokine concentrations reported are not normalized, and are that of swab resuspended in 1ml transport buffer. Levels are therefore significantly lower than true concentration on the penis surface.

### T cell isolation and characterization from foreskin tissue

T cells were isolated from foreskin samples obtained from Service Program participants as previously described [39]. Mononuclear cell counts were determined by trypan blue exclusion and 10-20x10^6^ cells (depending on yield) were stimulated with either 1ng/ml phorbol-12-myristate-13-acetate (PMA) and 1μg/ml ionomycin (both from Sigma; St. Louis, MO, USA) or vehicle (0.1% DMSO) in the presence of 5μg/ml Brefeldin A (GolgiPlug, BD Biosciences). After stimulation, samples were stained for CD3 (UCHT1), CD4 (RPA-T4), CD8 (SK1) and CCR5 (2D7/CCR5; all BD Biosciences). Samples for intracellular staining were permeabilized using BD Cytofix/Cytoperm solution and stained with TNFα (MAb11), IFNγ (B27; all BD Biosciences), or IL-17A (eBio64DEC17; eBiosciences). Samples were acquired using a FACSCalibur flow cytometer (BD Systems). Gating was performed as previously described [39] by investigators blinded to participant status and cytokine levels.

### Foreskin tissue immunochemistry studies

Proportions of T cell subsets were converted to absolute numbers per mm^2^ foreskin tissue using CD3 IHC as previously described [83]. Briefly, OCT-cryopreserved tissues were sectioned, fixed in 2% formaldehyde, and frozen for batch staining. Sections were stained with anti-CD3 antibody, followed by biotin-labeled secondary, Alkaline Phosphatase Streptavidin Labeling Reagent and Substrate Kit Vector Red (all Vector Labs, Burlingame, CA), and then counterstained with Mayer’s Hematoxylin (Fisher Scientific). The number of CD3+ T cells per mm^2^ of tissue for each patient was derived from the average of two biopsies taken from distal locations on the foreskin (median 6.10mm^2^ tissue/patient analyzed). Whole sections were scanned using the TissueScope 4000 (Huron Technologies, Waterloo, Canada) and image analysis software (Definiens, München, Germany) was used to delineate the apical edge of the epidermis to a depth of 300μm into the dermis (excluding artifacts or folds). CD3+ cells within this area were manually counted by a single investigator blinded to cytokine levels and participant status.

Neutrophil, Langerhans cell, and dermal dendritic cell density was assessed using immunofluorescence in a subset of men with high (n = 5) and low (n = 5) levels of coronal sulcus cytokines. Tissues cryopreserved in OCT were sectioned to 5μm using a Leica CM3050 cryostat (Leica Microsystems, Wetzlar, Germany), mounted on glass microscope slides, fixed for 7 minutes in ice-cold acetone, air-dried, and frozen at -80°C for batch staining. For staining, slides were thawed, permeabilized in PBS-Tween 20 for 20 minutes, and blocked using a streptavidin/biotin blocking kit (Vector Labs) and 10% normal rabbit serum. Neutrophils were visualized using biotin-labeled mouse anti-human CD15 antibody (eBiosciences) followed by DyLight 488 Streptavidin (Vector Labs) secondary. Dendritic cells were visualized with goat anti-human CD207 antibody (R&D Systems) and biotin-labeled mouse anti-human CD11c (eBiosciences). Slides were then washed and mounted using Vectashield HardSet Mounting Medium with DAPI Counterstain (Vector Labs), according to manufacturer’s instructions. Whole sections were scanned using the Zeiss Axioscan (Carl Zeiss Microscopy, Cambridge, UK) and image analysis software (Definiens) was used to delineate and quantify the epidermal and dermal tissue (excluding artifacts or folds). Definiens was then used to count cell populations using a threshold set by an investigator blinded to cytokine levels and participant status. CD15+ cells were counted within total foreskin area (median area analyzed = 4.06 mm^2^), CD207+ cells in the epidermal tissue (1.36 mm^2^), and CD11c+ cells within dermal tissue (3.39 mm^2^).

### Statistical analysis

We used Stata 13.1 for Mac (College Station, TX, USA) to conduct statistical analysis and Prism 5.0 (GraphPad Software; La Jolla, CA, USA) to construct graphs. Flow cytometry data was analyzed in FlowJo 9.8.2 (Treestar; Ashland, OR, USA). All tests two-sided with α = 0.05.

#### Statistical analysis: Coronal sulcus cytokines and HIV acquisition

To assess the relationship between coronal sulcus cytokine levels and risk of HIV acquisition, we performed a nested case-control study of men enrolled in the circumcision RCT. Our analysis was limited to those men who remained uncircumcised throughout the RCT, since circumcision is associated with a decreased risk of HIV [29] and cytokine changes (Fig 1). Cases consisted of 60 uncircumcised initially HIV-uninfected men who acquired HIV: 22 men seroconverted between baseline and 6 month’s follow-up, 17 between months 6 and 12, and 22 between months 12 and 24. Cytokine levels were analyzed in swabs collected at the participant’s last HIV-negative visit prior to seroconversion (therefore including 22 swabs from baseline visits, 17 swabs from month 6, and 21 swabs from month 12). Controls consisted of 120 men who remained persistently seronegative throughout the RCT, frequency-matched for visit with the seroconversion cases (i.e. 44 swabs from baseline visits, 34 swabs from month 6 visits, and 42 swabs from month 12 visits), to account for secular changes in cytokine levels over the trial period and to ensure no difference in storage time between case and control swabs. Demographics and sexual behaviors recorded at the time of swab collection were compared between cases and controls using Fisher's exact test. Because cytokine levels were generally low (below the assay LLOQ in at least 60% of swabs for all analytes), cytokine levels were dichotomized into “detectable” and “undetectable” based on the LLOQ for each analyte, and treated as binary exposures. In order to identify potential confounding variables (i.e., variables associated with both coronal sulcus cytokine detection and HIV acquisition), these same factors were also compared between men with detectable and undetectable cytokine levels using Fisher’s exact test.

Multivariate conditional logistic regression (matching on visit) was used to assess the association between the presence of each cytokine and the odds ratio (OR) and 95% confidence interval (CI) of HIV seroconversion relative to persistently seronegative controls. We used multivariate logistic regression models to estimate adjusted ORs (aOR) and 95% CI of HIV seroconversion associated with each coronal sulcus cytokine. Additional covariates in the adjusted models included occupation, marital status, number of sex partners, condom use, alcohol use, syphilis prevalence, and HSV-2 prevalence. To determine if the number of coronal sulcus cytokines detected was associated with a higher risk of HIV seroconversion, multivariate logistic regression was used treating number of cytokines detectable as a continuous variable and as a categorical variable (0, 1, or ≥2 cytokines present).

#### Statistical analysis: Impact of circumcision on coronal sulcus cytokines

To determine the impact of circumcision on coronal sulcus cytokines, we analyzed cytokine levels longitudinally (at enrolment and at months 6, 12, and 24) in men randomized to either circumcision (n = 80) or delayed circumcision (“uncircumcised”, n = 80) in the circumcision RCT. This sample size (n = 160) provided us with an 80% chance of detecting a 33% decrease in the prevalence of detectable IL-8 (α = 0.05, two-sided, assuming 65% prevalence of detectable IL-8 before circumcision). All men in this analysis were uncircumcised at the enrolment visit, and remained persistently HIV seronegative throughout the trial. Cytokine levels were dichotomized as “detectable” or “undetectable”. Enrolment demographics were compared between men randomized to immediate or delayed circumcision, and between men with detectable or undetectable cytokines, using Fisher’s exact test. Cytokine detection prevalence was compared between circumcised and uncircumcised men at each visit using Fisher’s exact test. For cytokines that differed between circumcised and uncircumcised men at follow-up visits (S4 Table), we used logistic regression models with generalized estimating equations (GEE) and robust variance estimators (unstructured correlation model) to determine if cytokine prevalence decreased at each follow-up visit compared to enrolment separately for each trial arm. Lastly, including men from both RCT arms, we used a logistic GEE model with an interaction terms for visit and assignment to determine if changes in cytokine detectability over time significantly differed by circumcision status.

#### Statistical analysis: Prepuce cytokines and foreskin T cell density

Cytokines were measured in coronal sulcus swabs from all 89 HIV-negative men enrolled though the Circumcision Service Program. During analysis IL-8 was treated as a continuous variable, with values below the LLOQ (5.6% of swabs) imputed as the value of the LLOQ (1.5pg/ml). MIG was less abundant, and thus treated as a binary variable, dichotomized at the LLOQ (0.3pg/ml). Concentration of IL-8 was correlated with the density of each cellular subset using simple linear regression. Densities of immune cell populations were compared between men with detectable and undetectable MIG, or between men with high and low cytokine levels, by the Mann-Whitney U test. Male and female partner cytokine levels were correlated using Spearman’s rank correlation coefficient.

## Supporting Information

S1 Table Participant demographics (case-control study of HIV seroconverters), stratified by presence of IL-8. (PDF)

S2 Table Participant demographics (case-control study of HIV seroconverters), stratified by presence of MIG. (PDF)

S3 Table Demographics of Rakai RCT participants (longitudinal study of circumcision). (PDF)

S4 Table Comparison of the frequency of detection of each cytokine between circumcised and uncircumcised men, performed cross-sectionally at each time point. (PDF)

S5 Table Demographic of participants enrolled through the Rakai Circumcision Service Program (n = 89). (PDF)

## References

[1] UNAIDS. Fact sheet: 2014 statistics 2015 [updated 24 August 2015; cited 2015 September 16 2015]. http://www.unaids.org/en/resources/documents/2015/20150714_factsheet.

[2] UNAIDS. Global Report: UNAIDS report on the global AIDS epidemic 2013. WHO Library Cataloguing-in-Publication Data: 2013.

[3] Boily MC, Baggaley RF, Wang L, Masse B, White RG, Hayes RJ, et al Heterosexual risk of HIV-1 infection per sexual act: systematic review and meta-analysis of observational studies. Lancet Infect Dis. 2009;9(2):118–29. Epub 2009/01/31. 10.1016/S1473-3099(09)70021-0 19179227 PMC4467783

[4] Hladik F, McElrath MJ. Setting the stage: host invasion by HIV. Nat Rev Immunol. 2008;8(6):447–57. Epub 2008/05/13. 10.1038/nri2302 18469831 PMC2587276

[5] Gregson S, Nyamukapa CA, Garnett GP, Mason PR, Zhuwau T, Carael M, et al Sexual mixing patterns and sex-differentials in teenage exposure to HIV infection in rural Zimbabwe. Lancet. 2002;359(9321):1896–903. Epub 2002/06/12. 10.1016/S0140-6736(02)08780-9 12057552

[6] Yi TJ, Shannon B, Prodger J, McKinnon L, Kaul R. Genital immunology and HIV susceptibility in young women. Am J Reprod Immunol. 2013;69 Suppl 1:74–9. 23157424 10.1111/aji.12035

[7] Kaul R, Cohen CR, Chege D, Yi TJ, Tharao W, McKinnon LR, et al Biological factors that may contribute to regional and racial disparities in HIV prevalence. Am J Reprod Immunol. 2011;65(3):317–24. Epub 2011/01/13. 10.1111/j.1600-0897.2010.00962.x 21223426

[8] Galvin SR, Cohen MS. The role of sexually transmitted diseases in HIV transmission. Nat Rev Microbiol. 2004;2(1):33–42. Epub 2004/03/24. 10.1038/nrmicro794 15035007

[9] Weiss HA, Quigley MA, Hayes RJ. Male circumcision and risk of HIV infection in sub-Saharan Africa: a systematic review and meta-analysis. Aids. 2000;14(15):2361–70. Epub 2000/11/23. 11089625 10.1097/00002030-200010200-00018

[10] Siegfried N, Muller M, Deeks JJ, Volmink J. Male circumcision for prevention of heterosexual acquisition of HIV in men. Cochrane Database Syst Rev. 2009;(2):CD003362. Epub 2009/04/17. 10.1002/14651858.CD003362.pub2 PMC11666075 19370585

[11] Stieh DJ, Maric D, Kelley ZL, Anderson MR, Hattaway HZ, Beilfuss BA, et al Vaginal challenge with an SIV-based dual reporter system reveals that infection can occur throughout the upper and lower female reproductive tract. PLoS Pathog. 2014;10(10):e1004440 10.1371/journal.ppat.1004440 25299616 PMC4192600

[12] Keele BF, Giorgi EE, Salazar-Gonzalez JF, Decker JM, Pham KT, Salazar MG, et al Identification and characterization of transmitted and early founder virus envelopes in primary HIV-1 infection. Proc Natl Acad Sci U S A. 2008;105(21):7552–7. Epub 2008/05/21. 10.1073/pnas.0802203105 18490657 PMC2387184

[13] Li Q, Estes JD, Schlievert PM, Duan L, Brosnahan AJ, Southern PJ, et al Glycerol monolaurate prevents mucosal SIV transmission. Nature. 2009;458(7241):1034–8. 10.1038/nature07831 19262509 PMC2785041

[14] Joag V, McKinnon LR, Liu J, Kidane ST, Yudin MH, Nyanga B, et al Identification of preferential CD4+ T cell targets of HIV infection in the cervix. Mucosal Immunol. 2016 9(1):1–12. 25872482 10.1038/mi.2015.28

[15] Ganor Y, Zhou Z, Tudor D, Schmitt A, Vacher-Lavenu MC, Gibault L, et al Within 1 h, HIV-1 uses viral synapses to enter efficiently the inner, but not outer, foreskin mucosa and engages Langerhans—T cell conjugates. Mucosal Immunology. 2010;3(5):506–22. 10.1038/mi.2010.32 20571487

[16] Zhang Z. Sexual Transmission and Propagation of SIV and HIV in Resting and Activated CD4+ T Cells. Science. 1999;286(5443):1353–7. 10558989 10.1126/science.286.5443.1353

[17] Zhang ZQ. Roles of substrate availability and infection of resting and activated CD4+ T cells in transmission and acute simian immunodeficiency virus infection. Proceedings of the National Academy of Sciences. 2004;101(15):5640–5. 10.1073/pnas.0308425101 PMC397458 15064398

[18] Saba E, Grivel JC, Vanpouille C, Brichacek B, Fitzgerald W, Margolis L, et al HIV-1 sexual transmission: early events of HIV-1 infection of human cervico-vaginal tissue in an optimized ex vivo model. Mucosal Immunol. 2010;3(3):280–90. Epub 2010/02/12. 10.1038/mi.2010.2 20147895 PMC3173980

[19] Meditz AL, Haas MK, Folkvord JM, Melander K, Young R, McCarter M, et al HLA-DR+ CD38+ CD4+ T lymphocytes have elevated CCR5 expression and produce the majority of R5-tropic HIV-1 RNA in vivo. J Virol. 2011;85(19):10189–200. Epub 2011/08/05. 10.1128/JVI.02529-10 21813616 PMC3196402

[20] Narimatsu R, Wolday D, Patterson BK. IL-8 increases transmission of HIV type 1 in cervical explant tissue. AIDS Res Hum Retroviruses. 2005;21(3):228–33. Epub 2005/03/30. 10.1089/aid.2005.21.228 15795529

[21] Tang S, Patterson B, Levy JA. Highly purified quiescent human peripheral blood CD4+ T cells are infectible by human immunodeficiency virus but do not release virus after activation. J Virol. 1995;69(9):5659–65. Epub 1995/09/01. 7637012 10.1128/jvi.69.9.5659-5665.1995 PMC189423

[22] Carnathan DG, Wetzel KS, Yu J, Lee ST, Johnson BA, Paiardini M, et al Activated CD4+CCR5+ T cells in the rectum predict increased SIV acquisition in SIVGag/Tat-vaccinated rhesus macaques. Proc Natl Acad Sci U S A. 2015;112(2):518–23. 10.1073/pnas.1407466112 25550504 PMC4299179

[23] Masson L, Passmore JA, Liebenberg LJ, Werner L, Baxter C, Arnold KB, et al Genital inflammation and the risk of HIV acquisition in women. Clin Infect Dis. 2015;61(2):260–9. 10.1093/cid/civ298 25900168 PMC4565995

[24] Arnold KB, Burgener A, Berse K, Dunphy L, Shahabi K, Abou M, et al Increased levels of inflammatory cytokines in the female reproductive tract are associated with altered expression of proteases, mucosal barrier proteins, and an influx of HIV susceptible target cells. Mucosal Immunol. 2016 9(1):194–205. 26104913 10.1038/mi.2015.51

[25] Freeman EE, Weiss HA, Glynn JR, Cross PL, Whitworth JA, Hayes RJ. Herpes simplex virus 2 infection increases HIV acquisition in men and women: systematic review and meta-analysis of longitudinal studies. AIDS. 2006;20(1):73–83. 16327322 10.1097/01.aids.0000198081.09337.a7

[26] Prodger JL, Gray R, Kigozi G, Nalugoda F, Galiwango R, Nehemiah K, et al Impact of asymptomatic Herpes simplex virus-2 infection on T cell phenotype and function in the foreskin. Aids. 2012;26(10):1319–22. Epub 2012/04/21. 10.1097/QAD.0b013e328354675c 22516874 PMC4241749

[27] Johnson KE, Redd AD, Quinn TC, Collinson-Streng AN, Cornish T, Kong X, et al Effects of HIV-1 and Herpes Simplex Virus Type 2 Infection on Lymphocyte and Dendritic Cell Density in Adult Foreskins from Rakai, Uganda. Journal of Infectious Diseases. 2011;203(5):602–9. 10.1093/infdis/jiq091 21220779 PMC3071278

[28] Bailey R, Moses S, Parker C, Agot K, Maclean I, Krieger J, et al Male circumcision for HIV prevention in young men in Kisumu, Kenya: a randomised controlled trial. The Lancet. 2007;369(9562):643–56. 10.1016/S0140-6736(07)60312-2 17321310

[29] Gray RH, Kigozi G, Serwadda D, Makumbi F, Watya S, Nalugoda F, et al Male circumcision for HIV prevention in men in Rakai, Uganda: a randomised trial. Lancet. 2007;369(9562):657–66. 10.1016/S0140-6736(07)60313-4 17321311

[30] Auvert B, Taljaard D, Lagarde E, Sobngwi-Tambekou J, Sitta R, Puren A. Randomized, controlled intervention trial of male circumcision for reduction of HIV infection risk: the ANRS 1265 Trial. PLoS Med. 2005;2(11):e298 10.1371/journal.pmed.0020298 16231970 PMC1262556

[31] Tobian AA, Serwadda D, Quinn TC, Kigozi G, Gravitt PE, Laeyendecker O, et al Male circumcision for the prevention of HSV-2 and HPV infections and syphilis. N Engl J Med. 2009;360(13):1298–309. Epub 2009/03/27. 10.1056/NEJMoa0802556 19321868 PMC2676895

[32] Liu CM, Hungate BA, Tobian AA, Serwadda D, Ravel J, Lester R, et al Male circumcision significantly reduces prevalence and load of genital anaerobic bacteria. MBio. 2013;4(2):e00076 Epub 2013/04/18. 10.1128/mBio.00076-13 23592260 PMC3634604

[33] Fischetti L, Barry SM, Hope TJ, Shattock RJ. HIV-1 infection of human penile explant tissue and protection by candidate microbicides. Aids. 2009;23(3):319–28. 10.1097/QAD.0b013e328321b778 19114867 PMC4349942

[34] Patterson BK, Landay A, Siegel JN, Flener Z, Pessis D, Chaviano A, et al Susceptibility to Human Immunodeficiency Virus-1 Infection of Human Foreskin and Cervical Tissue Grown in Explant Culture. American Journal of Pathology. 2002;161(3):867–73. 10.1016/S0002-9440(10)64247-2 12213715 PMC1867269

[35] Lemos MP, Lama JR, Karuna ST, Fong Y, Montano SM, Ganoza C, et al The inner foreskin of healthy males at risk of HIV infection harbors epithelial CD4+ CCR5+ cells and has features of an inflamed epidermal barrier. PLoS One. 2014;9(9):e108954 10.1371/journal.pone.0108954 25268493 PMC4182607

[36] Fahrbach KM, Barry SM, Ayehunie S, Lamore S, Klausner M, Hope TJ. Activated CD34-derived Langerhans cells mediate transinfection with human immunodeficiency virus. J Virol. 2007;81(13):6858–68. Epub 2007/04/20. 10.1128/JVI.02472-06 17442711 PMC1933306

[37] Izquierdo-Useros N, Blanco J, Erkizia I, Fernandez-Figueras MT, Borras FE, Naranjo-Gomez M, et al Maturation of blood-derived dendritic cells enhances human immunodeficiency virus type 1 capture and transmission. J Virol. 2007;81(14):7559–70. Epub 2007/05/04. 10.1128/JVI.02572-06 17475656 PMC1933337

[38] Wang JH, Janas AM, Olson WJ, Wu L. Functionally distinct transmission of human immunodeficiency virus type 1 mediated by immature and mature dendritic cells. J Virol. 2007;81(17):8933–43. Epub 2007/06/15. 10.1128/JVI.00878-07 17567699 PMC1951429

[39] Prodger JL, Gray R, Kigozi G, Nalugoda F, Galiwango R, Hirbod T, et al Foreskin T-cell subsets differ substantially from blood with respect to HIV co-receptor expression, inflammatory profile, and memory status. Mucosal Immunol. 2012;5(2):121–8. 10.1038/mi.2011.56 22089029 PMC3288185

[40] Tobian AA, Ssempijja V, Kigozi G, Oliver AE, Serwadda D, Makumbi F, et al Incident HIV and herpes simplex virus type 2 infection among men in Rakai, Uganda. Aids. 2009;23(12):1589–94. Epub 2009/05/29. 10.1097/QAD.0b013e32832d4042 19474649 PMC2715553

[41] Baggiolini M, Dewald B, Moser B. Human chemokines: an update. Annu Rev Immunol. 1997;15:675–705. 10.1146/annurev.immunol.15.1.675 9143704

[42] Hirbod T, Bailey RC, Agot K, Moses S, Ndinya-Achola J, Murugu R, et al Abundant expression of HIV target cells and C-type lectin receptors in the foreskin tissue of young Kenyan men. Am J Pathol. 2010;176(6):2798–805. Epub 2010/04/17. 10.2353/ajpath.2010.090926 20395432 PMC2877841

[43] Prodger JL, Hirbod T, Kigozi G, Nalugoda F, Reynolds SJ, Galiwango R, et al Immune correlates of HIV exposure without infection in foreskins of men from Rakai, Uganda. Mucosal Immunol. 2014;7(3):634–44. 10.1038/mi.2013.83 24150258 PMC3997757

[44] Stieh DJ, Matias E, Xu H, Fought AJ, Blanchard JL, Marx PA, et al Th17 Cells Are Preferentially Infected Very Early after Vaginal Transmission of SIV in Macaques. Cell Host Microbe. 2016;19(4):529–40. 10.1016/j.chom.2016.03.005 27078070 PMC4841252

[45] McKinnon LR, Kaul R. Quality and quantity: mucosal CD4+ T cells and HIV susceptibility. Curr Opin HIV AIDS. 2012;7(2):195–202. 10.1097/COH.0b013e3283504941 22314505

[46] Talbert-Slagle K, Atkins KE, Yan KK, Khurana E, Gerstein M, Bradley EH, et al Cellular superspreaders: an epidemiological perspective on HIV infection inside the body. PLoS Pathog. 2014;10(5):e1004092 10.1371/journal.ppat.1004092 24811311 PMC4014458

[47] Gosselin A, Monteiro P, Chomont N, Diaz-Griffero F, Said EA, Fonseca S, et al Peripheral blood CCR4+CCR6+ and CXCR3+CCR6+CD4+ T cells are highly permissive to HIV-1 infection. J Immunol. 2010;184(3):1604–16. 10.4049/jimmunol.0903058 20042588 PMC4321756

[48] Alvarez Y, Tuen M, Shen G, Nawaz F, Arthos J, Wolff MJ, et al Preferential HIV infection of CCR6+ Th17 cells is associated with higher virus receptor expression and lack of CCR5 ligands. J Virol. 2013. Epub 2013/08/02. 10.1128/JVI.01838-13 PMC3807416 23903844

[49] El Hed A, Khaitan A, Kozhaya L, Manel N, Daskalakis D, Borkowsky W, et al Susceptibility of Human Th17 Cells to Human Immunodeficiency Virus and Their Perturbation during Infection. The Journal of Infectious Diseases. 2010;201(6):843–54. 10.1086/651021 20144043 PMC2849315

[50] Dillon SM, Manuzak JA, Leone AK, Lee EJ, Rogers LM, McCarter MD, et al HIV-1 infection of human intestinal lamina propria CD4+ T cells in vitro is enhanced by exposure to commensal Escherichia coli. J Immunol. 2012;189(2):885–96. Epub 2012/06/13. 10.4049/jimmunol.1200681 22689879 PMC3395168

[51] Baggiolini M, Walz A, Kunkel SL. Neutrophil-activating peptide-1/interleukin 8, a novel cytokine that activates neutrophils. J Clin Invest. 1989;84(4):1045–9. 10.1172/JCI114265 2677047 PMC329758

[52] Leonard EJ, Yoshimura T. Neutrophil attractant/activation protein-1 (NAP-1 [interleukin-8]). Am J Respir Cell Mol Biol. 1990;2(6):479–86. 10.1165/ajrcmb/2.6.479 2189453

[53] Gillitzer R, Berger R, Mielke V, Muller C, Wolff K, Stingl G. Upper keratinocytes of psoriatic skin lesions express high levels of NAP-1/IL-8 mRNA in situ. J Invest Dermatol. 1991;97(1):73–9. 1711550 10.1111/1523-1747.ep12478128

[54] Baggiolini M, Clark-Lewis I. Interleukin-8, a chemotactic and inflammatory cytokine. FEBS Lett. 1992;307(1):97–101. 1639201 10.1016/0014-5793(92)80909-z

[55] Russo RC, Garcia CC, Teixeira MM, Amaral FA. The CXCL8/IL-8 chemokine family and its receptors in inflammatory diseases. Expert Rev Clin Immunol. 2014;10(5):593–619. 10.1586/1744666X.2014.894886 24678812

[56] Leonard EJ, Yoshimura T, Tanaka S, Raffeld M. Neutrophil recruitment by intradermally injected neutrophil attractant/activation protein-1. J Invest Dermatol. 1991;96(5):690–4. 2022877 10.1111/1523-1747.ep12470612

[57] Swensson O, Schubert C, Christophers E, Schroder JM. Inflammatory properties of neutrophil-activating protein-1/interleukin 8 (NAP-1/IL-8) in human skin: a light- and electronmicroscopic study. J Invest Dermatol. 1991;96(5):682–9. 2022875 10.1111/1523-1747.ep12470606

[58] Gasperini S, Marchi M, Calzetti F, Laudanna C, Vicentini L, Olsen H, et al Gene expression and production of the monokine induced by IFN-gamma (MIG), IFN-inducible T cell alpha chemoattractant (I-TAC), and IFN-gamma-inducible protein-10 (IP-10) chemokines by human neutrophils. J Immunol. 1999;162(8):4928–37. 10202039

[59] Tanida S, Yoshitomi H, Nishitani K, Ishikawa M, Kitaori T, Ito H, et al CCL20 produced in the cytokine network of rheumatoid arthritis recruits CCR6+ mononuclear cells and enhances the production of IL-6. Cytokine. 2009;47(2):112–8. 10.1016/j.cyto.2009.05.009 19535263

[60] Pelletier M, Maggi L, Micheletti A, Lazzeri E, Tamassia N, Costantini C, et al Evidence for a cross-talk between human neutrophils and Th17 cells. Blood. 2010;115(2):335–43. 10.1182/blood-2009-04-216085 19890092

[61] Sallusto F, Lanzavecchia A. Heterogeneity of CD4+ memory T cells: functional modules for tailored immunity. Eur J Immunol. 2009;39(8):2076–82. 10.1002/eji.200939722 19672903

[62] Ouyang W, Kolls JK, Zheng Y. The biological functions of T helper 17 cell effector cytokines in inflammation. Immunity. 2008;28(4):454–67. 10.1016/j.immuni.2008.03.004 18400188 PMC3424508

[63] Kato T, Kitagawa S. Regulation of neutrophil functions by proinflammatory cytokines. Int J Hematol. 2006;84(3):205–9. 10.1532/IJH97.06141 17050192

[64] Keller M, Spanou Z, Schaerli P, Britschgi M, Yawalkar N, Seitz M, et al T cell-regulated neutrophilic inflammation in autoinflammatory diseases. J Immunol. 2005;175(11):7678–86. 16301678 10.4049/jimmunol.175.11.7678

[65] Schaerli P, Britschgi M, Keller M, Steiner UC, Steinmann LS, Moser B, et al Characterization of human T cells that regulate neutrophilic skin inflammation. J Immunol. 2004;173(3):2151–8. 15265952 10.4049/jimmunol.173.3.2151

[66] Reich K, Papp KA, Matheson RT, Tu JH, Bissonnette R, Bourcier M, et al Evidence that a neutrophil-keratinocyte crosstalk is an early target of IL-17A inhibition in psoriasis. Exp Dermatol. 2015;24(7):529–35. 10.1111/exd.12710 25828362 PMC4676308

[67] Van Raemdonck K, Van den Steen PE, Liekens S, Van Damme J, Struyf S. CXCR3 ligands in disease and therapy. Cytokine Growth Factor Rev. 2015;26(3):311–27. 10.1016/j.cytogfr.2014.11.009 25498524

[68] Ganor Y, Bomsel M. HIV-1 transmission in the male genital tract. Am J Reprod Immunol. 2011;65(3):284–91. Epub 2010/12/01. 10.1111/j.1600-0897.2010.00933.x 21114566

[69] Zhou Z, Barry de Longchamps N, Schmitt A, Zerbib M, Vacher-Lavenu MC, Bomsel M, et al HIV-1 efficient entry in inner foreskin is mediated by elevated CCL5/RANTES that recruits T cells and fuels conjugate formation with Langerhans cells. PLoS Pathog. 2011;7(6):e1002100 Epub 2011/07/09. 10.1371/journal.ppat.1002100 21738469 PMC3128116

[70] Zhu J, Hladik F, Woodward A, Klock A, Peng T, Johnston C, et al Persistence of HIV-1 receptor-positive cells after HSV-2 reactivation is a potential mechanism for increased HIV-1 acquisition. Nat Med. 2009;15(8):886–92. Epub 2009/08/04. 10.1038/nm.2006 19648930 PMC2723183

[71] Bellan SE, Dushoff J, Galvani AP, Meyers LA. Reassessment of HIV-1 acute phase infectivity: accounting for heterogeneity and study design with simulated cohorts. PLoS Med. 2015;12(3):e1001801 10.1371/journal.pmed.1001801 25781323 PMC4363602

[72] Taha TE, Hoover DR, Dallabetta GA, Kumwenda NI, Mtimavalye LA, Yang LP, et al Bacterial vaginosis and disturbances of vaginal flora: association with increased acquisition of HIV. AIDS. 1998;12(13):1699–706. 9764791 10.1097/00002030-199813000-00019

[73] Myer L, Denny L, Telerant R, Souza M, Wright TC Jr., Kuhn L. Bacterial vaginosis and susceptibility to HIV infection in South African women: a nested case-control study. J Infect Dis. 2005;192(8):1372–80. 10.1086/462427 16170754

[74] Warren D, Klein RS, Sobel J, Kieke B Jr., Brown W, Schuman P, et al A multicenter study of bacterial vaginosis in women with or at risk for human immunodeficiency virus infection. Infect Dis Obstet Gynecol. 2001;9(3):133–41. 10.1155/S1064744901000242 11516061 PMC1784649

[75] Cohen CR, Lingappa JR, Baeten JM, Ngayo MO, Spiegel CA, Hong T, et al Bacterial vaginosis associated with increased risk of female-to-male HIV-1 transmission: a prospective cohort analysis among African couples. PLoS Med. 2012;9(6):e1001251 10.1371/journal.pmed.1001251 22745608 PMC3383741

[76] Rebbapragada A, Howe K, Wachihi C, Pettengell C, Sunderji S, Huibner S, et al Bacterial vaginosis in HIV-infected women induces reversible alterations in the cervical immune environment. J Acquir Immune Defic Syndr. 2008;49(5):520–2. 10.1097/QAI.0b013e318189a7ca 18989228

[77] Sturm-Ramirez K, Gaye-Diallo A, Eisen G, Mboup S, Kanki PJ. High levels of tumor necrosis factor-alpha and interleukin-1beta in bacterial vaginosis may increase susceptibility to human immunodeficiency virus. J Infect Dis. 2000;182(2):467–73. 10.1086/315713 10915077

[78] Ma CS, Chew GY, Simpson N, Priyadarshi A, Wong M, Grimbacher B, et al Deficiency of Th17 cells in hyper IgE syndrome due to mutations in STAT3. J Exp Med. 2008;205(7):1551–7. Epub 2008/07/02. 10.1084/jem.20080218 18591410 PMC2442632

[79] Puel A, Cypowyj S, Bustamante J, Wright JF, Liu L, Lim HK, et al Chronic mucocutaneous candidiasis in humans with inborn errors of interleukin-17 immunity. Science. 2011;332(6025):65–8. Epub 2011/02/26. 10.1126/science.1200439 21350122 PMC3070042

[80] Liu CM, Hungate BA, Tobian AA, Ravel J, Prodger JL, Serwadda D, et al Penile Microbiota and Female Partner Bacterial Vaginosis in Rakai, Uganda. MBio. 2015;6(3):e00589 10.1128/mBio.00589-15 26081632 PMC4471566

[81] de Jager W, Bourcier K, Rijkers GT, Prakken BJ, Seyfert-Margolis V. Prerequisites for cytokine measurements in clinical trials with multiplex immunoassays. BMC Immunol. 2009;10:52 10.1186/1471-2172-10-52 19785746 PMC2761376

[82] Gamiel JL, Tobian AAR, Laeyendecker OB, Reynolds SJ, Morrow RA, Serwadda D, et al Improved Performance of Enzyme-Linked Immunosorbent Assays and the Effect of Human Immunodeficiency Virus Coinfection on the Serologic Detection of Herpes Simplex Virus Type 2 in Rakai, Uganda. Clinical and Vaccine Immunology. 2008;15(5):888–90. 10.1128/CVI.00453-07 18321879 PMC2394849

[83] Prodger JL, Hirbod T, Gray R, Kigozi G, Nalugoda F, Galiwango R, et al HIV Infection in Uncircumcised Men Is Associated With Altered CD8 T-cell Function But Normal CD4 T-cell Numbers in the Foreskin. J Infect Dis. 2014;209(8):1185–94. 10.1093/infdis/jit644 24277744 PMC3969543

