# Supplementary material for: Chemokine Levels in the Penile Coronal Sulcus Correlate with HIV-1 Acquisition and Are Reduced by Male Circumcision in Rakai, Uganda
Source: PLoS Pathog. 2016 Nov 29;12(11):e1006025. doi: 10.1371/journal.ppat.1006025 (PMC5127584; doi:10.1371/journal.ppat.1006025)
Supplement: S1 Table — (PDF) [file ppat.1006025.s001.pdf]

Table S1. Participant demographics (case-control study of HIV seroconverters), stratified by presence of IL-8.

	Undetectable IL-8 (n=73)		Detectable IL-8 (n=107)		
	No.	Col %	No.	Col %	p-value
**Age**					
15-24	37	50.7	59	55.1	
25-29	13	17.8	23	21.5	0.464
30-49	23	31.5	25	23.4	
**Education**					
None	4	5.5	9	8.4	
Primary	52	71.2	71	66.4	0.693
Secondary+	17	23.3	27	25.2	
**Religion**					
Catholic	43	58.9	76	71.0	
Protestant	25	34.2	24	22.4	0.202
Other	5	6.8	7	6.5	
**Occupation**					
Substinance Agriculture	27	37.0	33	30.8	
Salaried Employment	6	8.2	3	2.8	
Trade/Shopkeeper	19	26.0	30	28.0	0.330
Student	7	9.6	17	15.9	
Other	14	19.2	24	22.4	
**Marital Status**					
Single	32	43.8	54	50.5	
Monogamous	33	45.2	42	39.3	0.843
Polygamous	4	5.5	5	4.7	
Separated	4	5.5	6	5.6	
**Sex partners**					
0	8	11.0	14	13.1	
1	42	57.5	58	54.2	0.875
2+	23	31.5	35	32.7	
**Condom use, if sexually active** (n=158)					
Not using	31	47.7	31	33.3	
Sometimes	18	27.7	42	45.2	**0.073**
Always	16	24.6	20	21.5	
**Genital washing**					
Less than daily	13	17.8	25	23.4	0.370
Daily or more	60	82.2	82	76.6	
**Alcohol use**	57	78.1	71	66.4	**0.088**
**Syphilis prevalence** (n=168)	2	2.9	4	4.0	0.718
**HSV-2 seroprevalence** (n=164)	25	37.9	36	36.7	0.991
