# Supplementary material for: Chemokine Levels in the Penile Coronal Sulcus Correlate with HIV-1 Acquisition and Are Reduced by Male Circumcision in Rakai, Uganda
Source: PLoS Pathog. 2016 Nov 29;12(11):e1006025. doi: 10.1371/journal.ppat.1006025 (PMC5127584; doi:10.1371/journal.ppat.1006025)
Supplement: S2 Table — (PDF) [file ppat.1006025.s002.pdf]

Table S2. Participant demographics (case-control study of HIV seroconverters), stratified by presence of MIG.

	Undetectable MIG (n=135)		Detectable MIG (n=45)		
	No.	Col %	No.	Col %	p-value
**Age**					
15-24	75	55.6	21	46.7	
25-29	24	17.8	12	26.7	0.400
30-49	36	26.7	12	26.7	
**Education**					
None	8	5.9	5	11.1	
Primary	97	71.9	26	57.8	0.190
Secondary+	30	22.2	14	31.1	
**Religion**					
Catholic	87	64.4	32	71.1	
Protestant	39	28.9	10	22.2	0.678
Other	9	6.7	3	6.7	
**Occupation**					
Substinance Agriculture	46	34.1	14	31.1	
Salaried Employment	8	5.9	1	2.2	
Trade/Shopkeeper	38	28.1	11	24.4	0.373
Student	19	14.1	5	11.1	
Other	24	17.8	14	31.1	
**Marital Status**					
Single	66	48.9	20	44.4	
Monogamous	56	41.5	19	42.2	0.894
Polygamous	6	4.4	3	6.7	
Separated	7	5.2	3	6.7	
**Sex partners**					
0	18	13.3	4	8.9	
1	77	57	23	51.1	0.390
2+	40	29.6	18	40.0	
**Condom use, if sexually active** (n=158)					
Not using	48	41	14	34.1	
Sometimes	44	37.6	16	39.0	0.677
Always	25	21.4	11	26.8	
	158				
**Genital washing**					
Less than daily	30	22.2	8	17.8	0.527
Daily or more	105	77.8	37	82.2	
**Alcohol use**	95	70.4	33	73.3	0.704
**Syphilis prevalence** (n=168)	4	3.2	2	4.7	0.660
**HSV-2 seroprevalence** (n=164)	48	39.3	13	31.0	0.673
