# Supplementary material for: Chemokine Levels in the Penile Coronal Sulcus Correlate with HIV-1 Acquisition and Are Reduced by Male Circumcision in Rakai, Uganda
Source: PLoS Pathog. 2016 Nov 29;12(11):e1006025. doi: 10.1371/journal.ppat.1006025 (PMC5127584; doi:10.1371/journal.ppat.1006025)
Supplement: S3 Table — (PDF) [file ppat.1006025.s003.pdf]

Table S3. Demographics of Rakai RCT participants (longitudinal study of circumcision).

	Circumcised (n=80)		Uncircumcised (n=80)		
	No.	Col %	No.	Col %	p-value
**Age**					
15-24	14	17.5	17	21.2	
25-29	25	31.2	28	35.0	0.627
30-49	41	51.2	35	43.8	
**Education**					
None	5	6.2	7	8.8	
Primary	61	76.2	55	68.8	0.564
Secondary+	14	17.5	18	22.5	
**Religion**					
Catholic	49	61.3	55	68.8	
Protestant	26	32.5	21	26.2	0.610
Other	5	6.2	4	5.0	
**Occupation**					
Subsistence Agriculture	42	52.5	40	50.0	
Salaried Employment	4	5.0	2	2.5	0.803
Trade/Shopkeeper	25	31.2	27	33.8	
Other	9	11.2	11	13.8	
**Marital Status**					
Monogamous	73	91.2	73	91.2	1.000
Polygamous	7	8.8	7	8.8	
**Sex partners**					
>=1	45	56.2	44	55.0	0.874
<=2	35	43.8	36	45.0	
**Condom use**					
Not using	49	61.3	50	62.5	
Sometimes	31	38.8	29	36.2	0.584
Always	0	0.0	1	1.2	
**Genital washing**					
Less than daily	15	18.8	16	20.3	0.811
Daily or more	65	81.2	63	79.7	
**Alcohol use**	60	75.0	59	73.8	0.856
**Syphilis prevalence** (n=155)	3	3.8	6	7.9	0.286
**HSV-2 seroprevalence** (n=157)	29	37.2	27	34.2	0.694
