# Supplementary material for: Chemokine Levels in the Penile Coronal Sulcus Correlate with HIV-1 Acquisition and Are Reduced by Male Circumcision in Rakai, Uganda
Source: PLoS Pathog. 2016 Nov 29;12(11):e1006025. doi: 10.1371/journal.ppat.1006025 (PMC5127584; doi:10.1371/journal.ppat.1006025)
Supplement: S4 Table — (PDF) [file ppat.1006025.s004.pdf]

Table S4. Comparison of the frequency of detection of each cytokine between circumcised and uncircumcised men, performed cross-sectionally at each time point.

	Percent Detectable (95% CI)				
	Uncircumcised (n=80)		Circumcised (n=80)		Prevalence Risk Ratio (95% CI)
	No.	%	No.	%	
**IL-8**					
Baseline	54	67.5	47	58.8	0.83 (0.61, 1.14)
Month 6	56	70.9	29	36.7	0.50 (0.36, 0.68)
Year 1	49	62.0	21	26.2	0.45 (0.32, 0.64)
Year 2	36	54.5	11	13.9	0.34 (0.22, 0.51)
**MIG**					
Baseline	11	13.8	15	18.8	1.19 (0.80, 1.77)
Month 6	15	19.0	7	8.9	0.60 (0.35, 1.04)
Year 1	13	16.5	8	10.0	0.73 (0.44, 1.22)
Year 2	7	10.6	9	11.4	1.04 (0.65, 1.66)
**MCP-1**					
Baseline	4	5.0	6	7.5	1.22 (0.67, 2.19)
Month 6	15	19.0	2	2.5	0.22 (0.09, 0.53)
Year 1	5	6.3	2	2.5	0.56 (0.21, 1.48)
Year 2	4	6.1	2	2.5	0.60 (0.23, 1.54)
**MIP-3a**					
Baseline	3	3.8	6	7.5	1.36 (0.76, 2.45)
Month 6	3	3.8	0	0.0	0
Year 1	3	3.8	1	1.2	0.49 (0.13, 1.92)
Year 2	1	1.5	0	0.0	0
**IL-1a**					
Baseline	4	5.0	4	5.0	1.00
Month 6	16	20.3	5	6.3	0.44 (0.24, 0.82)
Year 1	5	6.3	2	2.5	0.56 (0.21, 1.48)
Year 2	11	16.7	7	8.9	0.69 (0.41, 1.16)
**RANTES**					
Baseline	2	2.5	2	2.5	1.00
Month 6	3	3.8	0	0.0	0
Year 1	2	2.5	1	1.2	0.66 (0.16, 2.63)
Year 2	0	0.0	0	0.0	-
