# Supplementary material for: Chemokine Levels in the Penile Coronal Sulcus Correlate with HIV-1 Acquisition and Are Reduced by Male Circumcision in Rakai, Uganda
Source: PLoS Pathog. 2016 Nov 29;12(11):e1006025. doi: 10.1371/journal.ppat.1006025 (PMC5127584; doi:10.1371/journal.ppat.1006025)
Supplement: S5 Table — (PDF) [file ppat.1006025.s005.pdf]

Table S5. Demographic of participants enrolled through the Rakai Circumcision Service Program (n=89).

Participants (n=89)	No.	%
**Age**		
18-29	22	24.7
30-34	26	28.1
35-39	19	21.3
40+	23	25.8
**HSV-2 Seropositive**	39	44.8
**Self-Reported STI Symptoms (30 days)**		
uretheral dyscharge	0	0.0
balanitis/itching	3	3.4
ulcers	0	0.0
warts	0	0.0
**Condom use**		
never	69	77.5
sometimes	17	19.1
always	3	3.4
**Multiple sex partners last year**	23	25.8
**Current extramarital relationship**	13	14.6
